# Remission of screen-detected metabolic syndrome and its determinants: an observational study

**DOI:** 10.1186/1471-2458-12-778

**Published:** 2012-09-13

**Authors:** Corine den Engelsen, Kees J Gorter, Philippe L Salomé, Maureen van den Donk, Guy E Rutten

**Affiliations:** 1 Julius Center for Health Sciences and Primary Care, University Medical Center Utrecht, Huispostnr STR.6.131, P.O. Box 85500, 3508 GA, Utrecht, Netherlands; 2 Huisartsenzorg IJsselstein, locatie 't Steyn, Eiteren 15, 3401 PS, IJsselstein, Netherlands

**Keywords:** Metabolic syndrome, Abdominal obesity, Screening, Cardiovascular risk, Primary care

## Abstract

**Background:**

Early detection and treatment of the metabolic syndrome may prevent diabetes and cardiovascular disease. Our aim was to assess remission of the metabolic syndrome and its determinants after a population based screening without predefined intervention in the Netherlands.

**Methods:**

In 2006 we detected 406 metabolic syndrome cases (The National Cholesterol Education Program’s Adult Treatment Panel III (NCEP ATP III) definition) among apparently healthy individuals with an increased waist circumference. They received usual care in a primary care setting. After three years metabolic syndrome status was re-measured. We evaluated which baseline determinants were independently associated with remission.

**Results:**

The remission rate among the 194 participants was 53%. Baseline determinants independently associated with a remission were the presence of more than three metabolic syndrome components (OR 0.46) and higher levels of waist circumference (OR 0.91), blood pressure (OR 0.98) and fasting glucose (OR 0.60).

**Conclusions:**

In a population with screen-detected metabolic syndrome receiving usual care, more than half of the participants achieved a remission after three years. This positive result after a relatively simple strategy provides a solid basis for a nation-wide implementation. Not so much socio-demographic variables but a higher number and level of the metabolic syndrome components were predictors of a lower chance of remission. In such cases, primary care physicians should be extra alert.

## Background

The term ‘Metabolic Syndrome’ (MetS) refers to a clustering of cardiovascular risk factors, which is clinically relevant to identify people with a high cardiovascular risk. People with MetS have an increased risk of developing both type 2 diabetes and cardiovascular disease [1-4] and an up to fourfold increased risk of mortality from cardiovascular disease [2,3]. Early detection and adequate treatment can modify or even abolish the risk factors and thus prevent cardiovascular disease [5-8]. Identifying people who are less likely to achieve a remission is important, in order to give them more attention and to intensify their treatment. Several studies assessed remission of the MetS, all following a structured intervention. Such interventions are usually not in accordance with daily practice. Especially interventions performed in a randomized controlled trial setting often provide data in a clinical context that does not exist outside the trial itself, thereby limiting generalizability [9]. The more a follow-up trajectory fits in daily practice, the better a screening strategy can be implemented. However, data on remission of the MetS following usual care are scarce. The same applies to prognostic determinants for remission.

Our aim was to assess the remission of the MetS in individuals with screen-detected MetS and to determine which determinants at the time of diagnosis predict remission three years after screening, followed by usual care in a primary care setting.

## Methods

### Study design and population

Between October 2006 and April 2007 the ‘IJsselstein Screening for Central Obesity to detect metabolic syndrome’ was conducted in the city of IJsselstein among 12.000 individuals. The aim was to determine the feasibility and yield of a screening for MetS with self-measurement of waist circumference as the first step [10]. A total of 1.721 individuals with a self-measured increased waist circumference (≥ 88 cm in women, ≥ 102 cm in men) underwent all study procedures. They were aged 20–70 years and not previously diagnosed with diabetes, hypertension, dyslipidemia or cardiovascular disease. 473 of them were detected with MetS. Participants were advised to contact their primary care center for the results of the screening; no other intervention was performed. In case of detected cardiovascular risk factors, they should receive usual care according to the guidelines ‘Cardiovascular risk management’ [11] and ‘Type 2 Diabetes Mellitus’ [12] of the Dutch College of General Practitioners. Follow-up measurements took place three years after screening, in November and December 2009. 406 of the 473 participants with screen-detected MetS were invited for follow-up measurements. The other 67 were no longer eligible because of illness and death or because they were no longer visiting the same primary care physician. Three and six weeks after the invitation, reminders were sent to the non-responders.

The study was approved by the medical ethics committee of the University Medical Center Utrecht, the Netherlands. Written informed consent was obtained from all participants.

### Measurements

Both at screening and three years later at follow-up the same measurements were performed. In a physical examination body weight, height, waist circumference and blood pressure were measured. Venous blood samples were drawn after an overnight fast to determine fasting blood glucose, triglycerides and high-density lipoprotein (HDL) cholesterol. A detailed description of these measurements was described previously [10].

At baseline participants had completed a questionnaire to determine ethnicity, education level and lifestyle factors (smoking habits, physical activity). Education level was dichotomized, in which ‘high’ was defined as having completed a level of secondary education which permits entry to college. Smoking was regarded positive when the participant was currently smoking; in case of former or never smoking it was regarded negative. Physical activity was assessed using the validated SQUASH questionnaire [13], which measures habitual activities with respect to occupation, leisure time, household, transportation means, and other daily activities. The results were dichotomized based on the Dutch Standard Healthy Movement: a minimum of thirty minutes of moderately intensive exercise at least five days a week [14].

At follow-up, smoking status and level of physical activity were re-evaluated using the same questionnaire, in which participants were also questioned about consulting a dietician. Data about prescribed cardiovascular medication at the time of follow-up were collected from the physician’s electronic medical record. As a measure of follow-up behavior we used the number of visits to the practice nurse during the first year following screening, collected from the electronic medical record.

### Outcome measure

The MetS was defined according to The National Cholesterol Education Program’s Adult Treatment Panel III (NCEP ATP III) criteria, namely the presence of at least three of the following five components: increased waist circumference (≥ 88 cm in women, ≥ 102 cm in men), elevated blood pressure (systolic ≥ 130 and/or diastolic ≥ 85 mmHg), elevated triglycerides (≥ 1.7 mmol/L), elevated fasting glucose (≥ 6.1 mmol/L), and reduced HDL cholesterol (< 1.0 mmol/L in men, < 1.3 mmol/L in women) [15]. We defined remission as having less than three MetS components at follow-up, thereby no longer fulfilling the criteria for the presence of the MetS. We did not take the use of cardiovascular medication into account in the definition of remission.

### Determinants

Both socio-demographic and clinical characteristics were considered as potential baseline determinants of remission. We considered several determinants with regard to the follow-up period as potential confounders (consultation behavior, weight loss, change in level of physical activity and prescription of cardiovascular medication for a specific MetS component).

### Data analysis

Categorical variables are reported as numbers and percentages, continuous variables as means with standard deviations (SD) and non-normally distributed variables as median with interquartile range.

We checked for selection bias by testing for differences in baseline variables (gender, age, body mass index (BMI), waist circumference, HDL cholesterol, fasting glucose, triglycerides and blood pressure) between responders and non-responders, and between participants (responders willing to participate) and non-participants (responders who indicated not to be interested in follow-up measurements). Chi-square tests were used for categorical variables, independent samples t-tests for normally distributed continuous variables and Mann–Whitney tests for non-normally distributed continuous variables. The same tests were used to test for baseline differences between participants who achieved a remission and those who did not. Paired samples t-tests for normally distributed variables and the Wilcoxon signed-rank test for non-normally distributed variables were used to test for a significant change in mean risk factor levels between screening and follow-up. A p-value < 0.05 was considered significant.

For logistic regression analyses, triglyceride level was log transformed because of its skewed distribution. Waist circumference and HDL cholesterol were also transformed, taking into account the gender specific thresholds. The gender specific threshold was extracted from the values obtained in the examinations. The new variables indicate the absolute difference with the gender specific threshold.

Univariable and multivariable logistic regression analyses were performed to assess determinants of remission. All variables considered as potential baseline determinants (Table 3) were entered into model 1, except for BMI, because of the high correlation with waist circumference. In model 2 the follow-up determinants were included as co-variables. The models were reduced by means of backward selection, based on the p-value of the Wald test. The variables with the highest p-value were one by one removed, until the p-value for each variable became < 0.20. All MetS components as well as gender were kept in the model, regardless of their p-value. Analyses were performed using Statistical Package of Social Sciences (SPSS, version 17.0).

**Table 1:** Baseline characteristics of the population with screen-detected metabolic syndrome participating in follow-up measurements, divided into those who did and did not achieve a remission

**Baseline characteristics**		**Total group**	**Remission**		**P-value**
		**n = 194**	**Yes n = 103**	**No n = 91**	
Gender (% male)		104 (53.9)	53 (51.5)	51 (56.0)	0.52
Age (years)		49.0 ± 10.1	48.2 ± 10.9	49.8 ± 9.0	0.27
Ethnicity (% Western European)		187 (96.4)	100 (97.1)	87 (95.6)	0.58
Higher educated (%)		68 (35.1)	37 (35.9)	31 (34.1)	0.79
Smoking (%)		43 (22.2)	22 (21.4)	21 (23.1)	0.77
Physical activity (% adhering to Dutch Standard Healthy Movement^a^)		102 (53.4)	52 (51.5)	49 (53.8)	0.74
BMI (kg/m^2^)		30.1 ± 3.7	29.7 ± 3.5	30.7 ± 3.8	<0.05
Waist circumference (cm)	♂	109.9 ± 7.4	106.6 ± 5.5	113.3 ± 7.6	< 0.001
	♀	99.5 ± 8.8	98.4 ± 8.9	101.0 ± 8.7	0.18
Blood pressure (mmHg)	Systolic	143.5 ± 14.9	142.0 ± 12.5	145.3 ± 17.2	0.14
	Diastolic	88.0 ± 7.5	87.1 ± 7.3	89.1 ± 7.6	0.07
Triglycerides (mmol/L)		1.9 (1.6-2.3)	1.9 (1.6-2.3)	1.9 (1.7-2.5)	0.24
HDL cholesterol (mmol/L)	♂	1.1 ± 0.3	1.1 ± 0.3	1.1 ± 0.2	0.44
	♀	1.3 ± 0.3	1.4 ± 0.3	1.3 ± 0.3	0.04
Fasting glucose (mmol/L)		5.3 ± 1.1	5.0 ± 0.6	5.5 ± 1.5	0.01

Data are reported as means ± standard deviation or number (percentage). Non-normally distributed variables are reported as median (25^th^ - 75^th^ percentile).

^a^ a minimum of thirty minutes of moderately intensive exercise at least five days a week.

## Results

**Figure 1:**
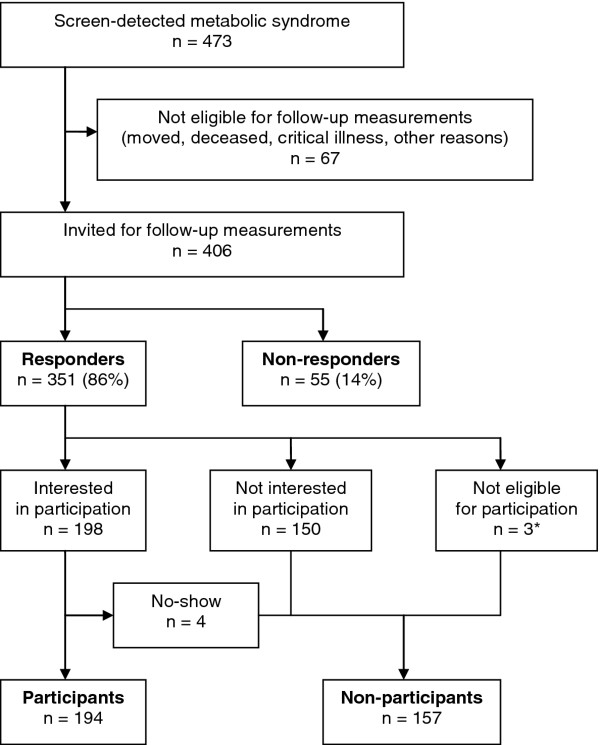
**Study flowchart. **Legend: * Reasons for exclusion: pregnancy (n = 1), illness (n = 1), stay abroad (n = 1).

The response rate was 86 %. 194 responders were willing to participate in follow-up examinations. The study flow chart is shown in Figure 1. 53.6 % of the participants were male and the mean age was 49.0 years (SD 10.1). Baseline characteristics are shown in Table 1.

Comparing the participants with the combined group of non-participants and non-responders showed that participants were older (49 versus 46 years, p < 0.01) and more often visited the practice nurse at least twice in the year following screening (69 versus 56 %, p < 0.01). Non-responders were significantly younger than responders (40 versus 49 years, p < 0.001) and more often of non-Western European origin (15 versus 4 %, p < 0.01) whereas responders more often than non-responders achieved the Dutch Standard Healthy Movement (42 versus 58 %, p = 0.03) and more often visited the practice nurse at least twice (65 versus 47 %, p = 0.01). Participants more often had a higher education level (35 versus 27 %, p < 0.05) than non-participants. However, they less often achieved the Dutch Standard Healthy Movement at baseline compared to non-participants (53 versus 64 %, p = 0.03).

Three years after screening a significant improvement in almost all MetS components was observed. However, the mean glucose level increased in both people with and without a remission of the MetS (Table 2). Of the 194 participants diagnosed with MetS, 103 (53.1 %) no longer fulfilled the MetS criteria according to their follow-up levels of the components. The participants who still fulfilled the MetS criteria at follow-up had a higher BMI and fasting glucose at baseline. Men not achieving a remission had a higher waist circumference, whereas women not achieving a remission had a significantly lower HDL cholesterol level at baseline (Table 1).

**Table 2:** Mean risk factor levels and mean change from baseline

**n = 194**		**2006**	**2009**	**Mean change from baseline**	**(95 % C.I.)**
Weight (kg)		93.1 ± 15.2	90.6 ± 16.1	−2.5 ± 6.4	(−3.4 ; -1.6)
BMI (kg/m^2^)		30.1 ± 3.7	29.2 ± 4.1	−0.9 ± 2.0	(−1.2 ; -0.6)
Waist circumference (cm)	♂	109.9 ± 7.4	106.2 ± 9.4	−3.6 ± 6.4	(−4.9 ; -2.4)
	♀	99.5 ± 8.8	95.9 ± 10.7	−3.6 ± 6.9	(−5.1 ; -2.1)
Blood pressure (mmHg)	Systolic	143.5 ± 14.9	135.4 ± 13.6	−8.1 ± 12.1	(−9.8 ; -6.4)
	Diastolic	88.0 ± 7.5	82.4 ± 7.7	−5.6 ± 7.7	(−6.7 ; -4.5)
Triglycerides (mmol/L)		1.9 (1.6-2.3)	1.8 (1.3-2.3)	−0.3 ± 1.1	(−0.4 ; -0.1)
HDL cholesterol (mmol/L)	♂	1.1 ± 0.3	1.2 ± 0.3	0.1 ± 0.1	(0.03 ; 0.08)
	♀	1.3 ± 0.3	1.4 ± 0.3	0.2 ± 0.2	(0.05 ; 0.14)
Fasting glucose (mmol/L)		5.3 ± 1.1	5.4 ± 0.7	0.2 ± 0.9	(0.0 ; 0.3)

Data are reported as means ± standard deviation or percentage. Non-normally distributed variables are reported as median (25^th^ - 75^th^ percentile).

At follow-up, 57 (29.4 %) participants received antihypertensive medication, 55 (28.4 %) received a statin and eight (4.1 %) were on blood glucose lowering medication. Fibrates or nicotinic acid were not prescribed. In 132 (68.0 %) participants, no medication for a specific MetS component was prescribed.

The presence of more than three MetS components and a higher waist circumference and fasting glucose were the baseline determinants significantly associated with a lower chance of remission in univariable regression analysis (Table 3).

**Table 3:** Associations of potential determinants with remission in univariable logistic regression analysis

		**n (%)**	**Remission rate (%)**	**OR**^**a**^	**(95 % C.I.)**	**P-value**
*Baseline determinants*						
Age (years)				0.98	(0.95 ; 1.01)	0.27
Gender	♂	104 (53.6)	51.0	Ref.		
	♀	90 (46.4)	55.6	1.20	(0.68 ; 2.12)	0.52
Ethnicity	Western European	187 (96.4)	53.5	Ref.		
	Other	7 (3.6)	42.9	0.65	(0.14 ; 3.00)	0.58
Higher educated	No	126 (64.9)	52.4	Ref.		
	Yes	68 (35.1)	54.4	1.09	(0.60 ; 1.96)	0.79
Smoking	No	151 (77.8)	53.6	Ref.		
	Yes	43 (22.2)	51.2	0.91	(0.46 ; 1.78)	0.77
Number of metabolic syndrome components	3	149 (76.8)	59.7	Ref.		
	≥ 4	45 (23.2)	31.1	0.30	(0.15 ; 0.62)	<0.01
BMI (kg/m^2^)				0.92	(0.85 ; 1.00)	0.05
HDL cholesterol (mmol/L)				2.64	(0.97 ; 7.18)	0.06
Waist circumference (cm)				0.93	(0.89 ; 0.97)	<0.001
Blood pressure (mmHg)	Systolic			0.99	(0.97 ; 1.01)	0.14
	Diastolic			0.96	(0.93 ; 1.00)	0.07
Triglycerides (mmol/L)				0.57	(0.29 ; 1.11)	0.10
Fasting glucose (mmol/L)				0.55	(0.36 ; 0.85)	<0.01
*Follow-up determinants*						
≥ 2 consultations with practice nurse	No	60 (30.9)	51.7	Ref.		
	Yes	134 (69.1)	53.7	1.09	(0.59 ; 2.00)	0.07
Cardiovascular medication, specific for metabolic syndrome component	No	132 (68.0)	56.1	Ref.		
	Yes	62 (32.0)	46.8	0.69	(0.38 ; 1.26)	0.23
Consultation with dietician	No	133 (68.6)	55.6	Ref.		
	Yes	61 (31.4)	47.5	0.72	(0.39 ; 1.33)	0.30
Change in physical activity level (hours/week)				1.00	(0.98 ; 1.01)	0.48
Weight loss (kg)				1.14	(1.07 ; 1.20)	<0.001

^a^ Odds ratio per one-unit change.

The odds ratios for the variables that were independently associated with remission (p < 0.20) are shown in Table 4. When evaluating only baseline determinants, age, a higher waist circumference and glucose, a lower HDL cholesterol and the presence of more than three MetS components were independently associated with a lower chance of remission. When adding follow-up determinants as co-variables, age and HDL cholesterol no longer showed an independent association with remission, whereas weight loss during follow-up did. Each kilogram of weight loss increased the likelihood of achieving a remission 1.2 times.

**Table 4:** Determinants independently associated (p < 0.20) with remission in multivariable logistic regression analysis

	**Model 1**					**Model 2**				
	**OR**^**a**^	**(95 % C.I.)**	**OR**^**b**^	**(95 % C.I.)**	**P-value**	**OR**^**a**^	**(95 % C.I.)**	**OR**^**b**^	**(95 % C.I.)**	**P-value**
Age (years)	0.98	(0.94 ; 1.01)			0.18					
Gender (female)	1.37	(0.69 ; 2.73)			0.37	1.23	(0.58 ; 2.61)			0.59
Waist circumference (cm)	0.93	(0.89 ; 0.97)	0.56	(0.39 ; 0.81)	<0.01	0.91	(0.87 ; 0.96)	0.46	(0.30 ; 0.71)	<0.01
HDL cholesterol (mmol/L)	2.52	(0.73 ; 8.69)	1.31	(0.91 ; 1.90)	0.14	1.69	(0.48 ; 5.89)	1.17	(0.81 ; 1.69)	0.41
Fasting glucose (mmol/L)	0.68	(0.43 ; 1.10)	0.65	(0.38 ; 1.11)	0.11	0.60	(0.36 ; 1.00)	0.56	(0.31 ; 1.00)	0.05
Triglycerides (mmol/L)	0.94	(0.41 ; 2.16)	0.97	(0.68 ; 1.40)	0.88	0.64	(0.27 ; 1.55)	0.82	(0.56 ; 1.21)	0.32
Systolic blood pressure (mmHg)	0.99	(0.97 ; 1.01)	0.84	(0.60 ; 1.18)	0.32	0.98	(0.95 ; 1.00)	0.70	(0.49 ; 1.01)	0.05
≥ 4 metabolic syndrome components	0.48	(0.20 ; 1.13)			0.09	0.46	(0.18 ; 1.19)			0.11
Weight loss (kg)						1.18	(1.10 ; 1.27)			<0.001

Model 1: only baseline determinants were entered into the model. The metabolic syndrome components and gender were kept in the model, regardless of their p-value.

Model 2: both baseline and follow-up determinants were entered into the model. The metabolic syndrome components and gender were kept in the model, regardless of their p-value.

^a^ Odds ratio per one-unit change.

^b^ Odds ratio per standard deviation increase.

## Discussion

We performed a screening in primary care among apparently healthy individuals, to detect new MetS cases. Participants were only advised to contact their primary care center for the results of the screening. Upon contacting they were expected to receive usual care according to existing guidelines; no specific intervention was designed. More than half of the participants detected with MetS at screening no longer fulfilled the metabolic syndrome criteria three years later. The presence of more than three MetS components and a higher waist circumference, glucose level and systolic blood pressure were independently associated with a lower chance of remission.

Several studies have assessed the remission of MetS, all after an intervention. These interventions include diet (remission rate 21-61 %) [16-18], exercise programs (remission rate 42-58 %) [19,20], combinations of both (remission rate 67 %) [21], bariatric surgery (remission rate up to 95 %) [22-24] and medication such as metformin, fenofibrate and orlistat (remission rate 23-44 %) [25,26]. However, data about remission of the MetS without predefined intervention unless the advice to contact the primary care center are scarce. Two randomized controlled trials reported remission rates for their control groups of 9 and 18 % [26,27]. Our higher remission rate might be explained by the intensity of our usual care, which was presumably higher than control group care as described by Orchard et al. and Bo et al. In these studies participants received lifestyle recommendations once, while the majority of our participants was seen several times by the practice nurse. The remission rate of 52 % observed in a prospective study more closely resembled our remission rate, which could be explained by an intervention more in agreement with our usual care [28]. However the follow-up period was only six months; it is uncertain what would have happened after this relatively short intervention period.

In our study, not only individuals with diabetes or cardiovascular disease were excluded. Individuals were only eligible for screening if they were not previously diagnosed with hypertension or dyslipidemia and did not use antihypertensive, blood glucose lowering or cholesterol lowering medication: they were apparently healthy. The shock of suddenly being diagnosed with several risk factors might have been an extra impulse to change lifestyle, which might have contributed to the high remission rate. Two studies compared baseline characteristics between participants who did and did not achieve a remission [17,19]. As in our study, the participants who did not achieve a remission had worse baseline values than the participants who did achieve a remission. This might be explained by the fact that achieving a remission is more difficult for participants with higher risk factor levels. Baseline HDL cholesterol level was not associated with remission, in contrast to baseline levels of glucose, waist circumference and systolic blood pressure, where lower baseline levels were associated with a higher chance of remission. HDL dysfunction could be an underlying reason for the absence of an association between baseline HDL cholesterol level and remission. Dysfunctional HDL particles lose their anti-inflammatory and atheroprotective properties. This condition is closely linked to obesity and to inflammation and might be more prevalent among people with high HDL concentrations. In Western populations, individuals with glucose intolerance or those at risk for cardiometabolic disease – people with MetS – could be affected by impaired function of HDL [29,30].

### Study limitations and strengths

This is the first study assessing remission of MetS after screening among apparently healthy people, without a predefined intervention program. The follow-up period of three years provides us with intermediate-term results. A shorter follow-up period might give too optimistic remission rates, because treatment effects and lifestyle changes tend to level off over time. Both socio-economic and demographic, biochemical and clinical variables were taken into account, as well as lifestyle factors. Our data on the prescription of cardiovascular medication were based on prescription according to the electronic medical record of the primary care physician. Actual use of the prescribed medication might be lower, since medication compliance, especially in primary prevention, is not optimal [31-33].

According to the NCEP ATP III criteria, when a patient is on drug treatment for a specific MetS component (antihypertensive drug treatment, blood glucose lowering treatment or treatment with a fibrate or nicotinic acid for a reduced HDL cholesterol or an increased triglyceride level) this component should be regarded as present, irrespective of the actual level of the component [15]. This means that for example the blood pressure component will be regarded as positive as long as someone is on antihypertensive drug treatment, despite perfect blood pressure levels and thereby a reduced cardiovascular risk. Therefore, to gain insight into the actual reduction in cardiovascular risk achieved by remission, we chose not to take drug treatment for a specific MetS component into our definition of remission. In fact, the definition of the MetS provides a good screening tool to detect people with a high cardiovascular risk, but the current NCEP ATP III definition is less suitable for evaluating the effect of an intervention on changes in risk. If we had taken prescription of medication for a specific MetS component into account in our definition our remission rate would have been 49.0 %.

The overall response rate was good, although a substantial amount of the responders indicated not to be interested in participating in follow-up. This resulted in a relatively small study population. One might assume that people willing to participate were the more motivated patients, leading to potential bias for the generalization of the results. Indeed the participants more often entered a follow-up regimen after screening than the non-participants and non-responders. Whether their higher motivation also has led to a higher remission rate is questionable. However, we have to take into account this potential bias in interpreting the results. Especially younger people were less interested in follow-up measurements. At the initial screening, it were also the younger subgroups in which an invitation reminder was necessary to get a sufficient response. Apparently first it takes more effort to involve younger people in screening, and then we gain less insight into the impact of the screening for their cardiovascular health. It would be interesting to know the remission rate among the younger non-participants, since half of the people detected with the MetS were younger than 50 years [10]. Age, however, was not significantly associated with achieving a remission in multivariable analysis.

## Conclusions

Screening with self-measurement of waist circumference as a first step detected 473 MetS cases among apparently healthy people. Among those who were willing to participate in follow-up examinations, 53 % no longer had the MetS after three years. This positive result after a relatively simple strategy provides a solid basis for a nation-wide implementation. However, people < 50 years seem less willing to participate in screening and follow-up, a finding that should be taken into account for future screening strategies. Not so much socio-demographic variables but a higher level of the MetS components and the presence of more than three components were baseline predictors of a lower chance of remission. In such cases, primary care physicians should be extra alert.

## Abbreviations

Mets: Metabolic Syndrome; NCEP ATP III: The National Cholesterol Education Program’s Adult Treatment Panel III; BMI: Body Mass Index; HDL: Cholesterol High-Density Lipoprotein Cholesterol.

## Competing interests

The authors declare that they have no competing interests.

## Authors’ contributions

CdE researched the data, performed the statistical analyses and wrote the manuscript. KJG, PLS, MvdD and GER contributed to the discussion. All authors were involved in the design of the study and read and approved the final manuscript.

## Pre-publication history

The pre-publication history for this paper can be accessed here:

http://www.biomedcentral.com/1471-2458/12/778/prepub
